# A Greedy Scanning Data Collection Strategy for Large-Scale Wireless Sensor Networks with a Mobile Sink

**DOI:** 10.3390/s16091432

**Published:** 2016-09-06

**Authors:** Chuan Zhu, Sai Zhang, Guangjie Han, Jinfang Jiang, Joel J. P. C. Rodrigues

**Affiliations:** 1 Department of Information and Communication Systems, Hohai University, Changzhou 213000, China; dr.river.zhu@gmail.com (C.Z.); zhangsai1991@gmail.com (S.Z.); jiangjinfang@hhu.edu.cn (J.J.); 2 National Institute of Telecommunications (Inatel), Minas Gerais 30000-000, Brazil; joeljr@ieee.org; 3 The Instituto de Telecomunicações, Universidade da Beira Interior, Covilhã 6200-062, Portugal; 4 The University ITMO, St. Petersburg 190000, Russia

**Keywords:** wireless sensor networks, data collection, mobile sink, greedy scanning

## Abstract

Mobile sink is widely used for data collection in wireless sensor networks. It can avoid ‘hot spot’ problems but energy consumption caused by multihop transmission is still inefficient in real-time application scenarios. In this paper, a greedy scanning data collection strategy (GSDCS) is proposed, and we focus on how to reduce routing energy consumption by shortening total length of routing paths. We propose that the mobile sink adjusts its trajectory dynamically according to the changes of network, instead of predetermined trajectory or random walk. Next, the mobile sink determines which area has more source nodes, then it moves toward this area. The benefit of GSDCS is that most source nodes are no longer needed to upload sensory data for long distances. Especially in event-driven application scenarios, when event area changes, the mobile sink could arrive at the new event area where most source nodes are located currently. Hence energy can be saved. Analytical and simulation results show that compared with existing work, our GSDCS has a better performance in specific application scenarios.

## 1. Introduction

With the development of wireless communication technologies, wireless sensor networks (WSNs) have been widely used in various applications, e.g., industry monitoring [1,2], scientific data collection [3,4,5], target tracking [6], military surveillance, and even underwater environment [7]. In these applications, the sensory data needs to be uploaded to the sink quickly and accurately; thus, data collection plays an important role in WSNs. In most of application scenarios, energy of sensor node is limited and can not be replenished [8,9,10], although some charging algorithms are proposed recently [11,12]. Hence, it is significant to economize energy consumption in data collection. Traditional methods of data collection are to utilize static sink or base station [13]. These methods always lead to “hot spot” problem. Hence, mobile sinks are introduced to solve this problem. Recent researches reveal that using the mobile property of sink is more promising for energy efficient data collection. In these researches, vehicles or aircrafts, which are equipped with wireless communication device, can move to the source nodes and collect data from them directly. It results in energy consumption balanced of the whole network and the lifetime of networks prolonged. In some application scenarios, nodes in WSNs may not be fully connected because of the presence of dead nodes or obstacles. Appointing a mobile sink to move into each unconnected region to collect data is a feasible solution. In this way, more information can be collected [14].

However, data collection using a mobile sink introduces new challenges for WSNs. One challenge is how to update the current location of sink with lower overheads and route data to it. The mobile sink can collect data in non-real time or real-time. In non-real time data collection, data is routed and buffered at certain nodes before it is collected by a periodically visiting mobile sink. It results in a high latency of data and the tendency of nodes’ memory overflow. In real-time data collection, the mobile sink moves around, and data is routed to the location of mobile sink, which requires nodes to obtain the current sink location [15]. It involves high control overheads for location updating and routing hand-offs. In this paper, we focus on real-time data collection using a mobile sink.

Another challenge is how to set a relative optimal mobility pattern for the mobile sink. There are three main mobility patterns: trajectory with dynamic adjustment, random walking and predetermined trajectory. The performance of a mobility pattern is judged by the following two parameters: energy consumption efficient, and network lifetime.

Based on the analysis mentioned above, we propose a greedy scanning data collection strategy for large-scale WSNs with a mobile sink. Our contributions are the following:

- Sink Location Local Updating Method: In our data collection strategy, the network is divided into virtual grids. When the mobile sink moves from one grid to another, only a few of the grids need to update the location of mobile sink. Sensory data from the whole network can be routed to the sink timely.
- Greedy Scanning Mobility Pattern: The mobile sink moves like a scanning curve in the screen of radar. According to the sum of data received from each direction, the mobile sink makes the decision to move to the area with more sensory data.

The remainder of this paper is organized as follows. Firstly, the related work of data collection schemes with a mobile sink is introduced in Section 2. The detail of GSDCS is described in Section 3. The simulation results and performance evaluations are given in Section 4. Finally, Section 5 concludes the paper.

## 2. Related Works

### 2.1. Overview

**Figure 1:**
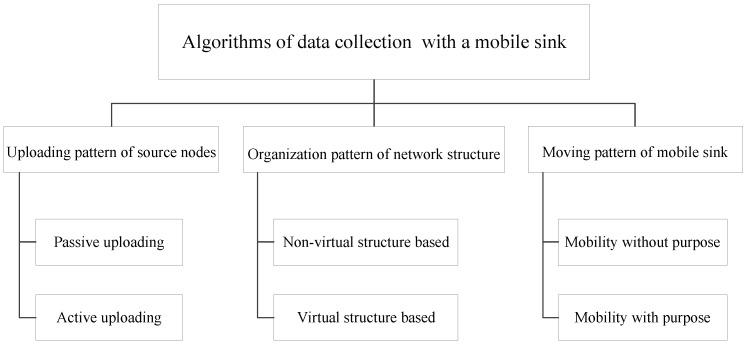
Categories of data collection algorithms with a mobile sink.

In recent years, data collection with a mobile sink has been widely concerned. Many algorithms have been proposed in this field. We can classify these algorithms into the following three aspects, which are illustrated as Figure 1.

(1) Uploading pattern of source nodes;
(2) Organization pattern of network structure;
(3) Moving pattern of mobile sink.

### 2.2. Uploading Pattern of Source Nodes

In the uploading pattern of source nodes, all algorithms can be further classified into passive uploading and active uploading. In the passive uploading pattern, source nodes do not upload the data immediately. Sensory data is buffered in some appointed nodes, which keep the buffered data until a mobile sink is reached [3,4,16,17,18,19,20,21,22,23]. In contrast, in the active uploading pattern, source nodes upload sensory data to the sink by multi-hop as soon as they sense the data [14,24,25,26,27,28,29].

#### 2.2.1. Passive Uploading Pattern

In the passive uploading pattern, the mobile sink needs to traverse in the network to collect data by short range communication. To minimize energy consumption, the mobile sink needs to visit each sensor node. That means each data packet can be transmitted to the sink directly. The energy used for packets relaying is saved in this way. However, the limited velocity of the mobile sink may lead to unacceptable time delay. Moreover, limited memory space may also lead to overflow. To overcome these problems, the idea of cluster is proposed. Sensory data is transmitted to the cluster head node in each cluster. The mobile sink just needs to visit these cluster heads. This approach is associated with a trade-off between energy consumption, time latency, and memory space usage rate.

In [3], Zhao et al. propose the bounded relay hop mobile data gathering (BRH-MDG) algorithm. It exploits a balance between the relay hop count of each cluster and the moving tour length of the mobile sink. This balance gets a trade-off between energy consumption and data gathering latency. In this algorithm, a subset of sensor nodes are selected as polling points (PPs). They buffer local sensory data and upload the data to the mobile sink when it arrives. The main point of this paper is that any data packet relay to corresponding PPs is bounded within a given number of hops.

BRH-MDG can reduce latency, but it does not consider the deadline of sensory data. Hence, a deadline is introduced in [16]. Weighted rendezvous planning (WRP) is proposed by Salarian et al. in [16]. This algorithm is mainly used in delay-sensitive applications. This means that all sensory data has a deadline. Mobile sink only visits rendezvous points (RPs), and other nodes upload sensory data to RPs. Different from BRH-MDG, WRP does not need to have an uploading bound and a shortest traverse path. WRP just needs to visit more RPs in a constraint time. Then energy can be saved. In an initial situation, the first RP is the location of the sink. There are some rounds in WRP. In each round, each node calculates a weight value. The weight value is the multiplication of packets number relayed and hop distance to the closest RP. The sensor node with the highest weight value is chosen as an RP. This RP is added in a traveling path. Sink judges whether the moving time of travel path is beyond the constraint time. If the moving time is still in the constraint time, the next round will be started. The calculation of weight value can improve the performance of data collection. However, the residual energy of node is not added in calculation. Hence some nodes with a higher weight value may have a lower residual energy. It affects the lifetime and the balance of network.

Although these algorithms can reduce latency to some degree, the latency of uploading is still hard to accept in an application where fresh data is very important. The complexity of planning tour is always high. Hence the pattern of passive uploading is not suitable for delay-sensitive application scenarios.

#### 2.2.2. Active Uploading Pattern

In the active uploading case, sensory data are uploaded to sink by multi-hop as soon as sensory data is sensed.

In [14], Zhu et al. propose Binary-Tree based Data Gathering Scheme (BTDGS). In this algorithm, a virtual binary-tree infrastructure is established. The whole network area is divided into many small sectors. There is a head node in each sector to collect data from this sector. The mobile sink moves along the boundary of network to collect data. Sensor nodes relay data packages in a greedy manner. When source nodes get data, they send the data toward the center area. That is because nodes in center area hold the position of sink. On the process of relaying to center area, the direction of relaying will change to sink upon meeting a node which has sink’s position. Then sensory data is routed to sink. However, the way of updating sink’s location is complexity, and sensory data is always routed to the center area firstly to find sink’s location. In this way, the route path becomes longer and more energy is consumed.

VGDRA [24], proposed by Khan et al., decreases the complexity of sink location updating than BTDGS. In VGDRA, the network is divided into virtual grids, and the number of virtual grids is decided by the number of sensor nodes. Each grid has a head node. When nodes get sensory data, they transmit data to their own head node immediately. The head nodes can communicate with each other directly, and data is relayed by head nodes. That means head nodes form the routing path. Sink moves around the network boundary with a constant speed. When sink is closer to a different grid, the formed routing path changes according to rule. The difference between VGDRA with BTDGS is that the sensory data can be routed by a formed routing path in VGDRA. When nodes sensed data, they do not need to take time to search for the location of the sink. However, another problem appears. As the sink moves along the network boundary to collect data, sensory data is all routed to the nodes which are in boundary grids. These nodes consume energy fast, and the ‘hot spots’ problem occurs.

Algorithms mentioned above adopt active uploading to achieve lower latency, as many application scenarios need fresh data. However, there are also some challenges with active uploading. The first challenge is how to update sink’s location quickly with lower energy consumption when sink is moving. The second challenge is that nodes around the sink always consume energy quickly. This may leads to the ‘hot spots’ problem.

### 2.3. Organization Pattern of Network Structure

According to organization pattern of network structure, these algorithms are classified into two categories: non-virtual structure based [16,25,30] and virtual structure based [14,20,24,26,27,28,29,31,32,33,34,35].

#### 2.3.1. Non-Virtual Structure Based Pattern

Some algorithms collect data without virtual structure. In [30], Han et al. propose the minimum Wiener index spanning tree (MWST). This algorithm can get a routing topology which is suitable for WSNs with mobile sink. As finding a spanning tree with the minimum Wiener index from a weighted graph is NP-hard, the authors propose a new way to solve this problem. They find that the minimum spanning tree (MST) has the Wiener index as close as that of the MWST through extensive experiments. The algorithm to find the MST from a given graph has low time complexity. Hence, the authors choose the Wiener index of the minimum spanning tree of a given graph as the initial upper bound. This algorithm provides a way to establish routing path more efficient without virtual structure, however the problem is that this algorithm does not give the way to find the current location of sink when the sink is moving.

In [25], Shin et al. propose a milestone-based predictive routing protocol which solves the problem mentioned in MWST. The algorithm consists of three parts. The first part is estimation of mobile sink’s future location. The mobile sink regularly broadcasts its updated location, calculated by the Formula (1), to its neighboring nodes. The $\vec{Sink}$ represents the mobile sink’s current moving speed and direction. If the mobile sink changes its direction, it estimates its future location by using the Formula (2). The $t_{c}$ is the current time and $t_{l}$ is the time when the sink has changed its moving direction recently.

(1) $$Sink_{updated}\left(X,Y\right)=Sink_{current}\left(X,Y\right)+\vec{Sink}$$

(2) $$Sink_{estimated}\left(X,Y\right)=Sink_{current}\left(X,Y\right)+\vec{Sink}\times \left(t_{c}-t_{l}\right)$$

The second part is about milestone nodes and the way to spread sink’s location information. This part introduces the concept of a milestone node. Its duty is to spread the estimated sink’s future location to the nodes located in the vicinity of the recent trail of the mobile sink. When sink changes its direction, it will choose a new milestone node. The third part is routing strategy. Sensory data is relayed to sink according to these milestone nodes. This algorithm gives the way to find current location of sink, and milestone nodes is the tool. However, the problem is that too many milestone nodes are needed when sink moves longer. The data routing path will be longer and longer, and control packets of routing path consume much energy. So the way to find current location of sink is always inefficient.

According to the algorithms mentioned above, we can find that the network without virtual structure always has higher complexity in routing data and sink location discovery.

#### 2.3.2. Virtual Structure Based Pattern

Many algorithms adopt virtual structure to collect data. TTDD [26], proposed by Luo et al., adopts square virtual grid structure to collect data. When a source node appears, it sponsors the establishment of the virtual grid structure. The source node is appointed as a crossing point of the virtual structure. It relays establishment information to all other crossing points, called dissemination points. The nodes which are closest to the virtual crossing points are responsible for the crossing points. As each source node establishes its own virtual structure, that means the virtual structure will be reestablished for many times. In this way, excessive energy is consumed. In [24], VGDRA can avoid the energy consumption of reestablishment. The establishment of virtual structure has nothing to do with source node in this algorithm. VGDRA partitions target area into uniform square cell. The number of square cell is based on the total number of sensor nodes. VGDRA adopts the heuristics used in LEACH [36], TEEN [37], and APTEEN [15] which consider 5% of the total number of sensor nodes. The node which is closer to center of virtual cell and has more residual energy will be chosen as a head node in each grid cell. The sensory data is relayed among head nodes to the mobile sink. Although the reestablishment is avoided, communication range and side length of target area are not taken into consideration.

In the algorithms with virtual structure, nodes are organized by corresponding virtual cell and head node. Sensory data is easy to be routed to sink by virtual structure when sink is moving. However, the attendant challenge is that the maintenance of some virtual structure is complex.

### 2.4. Moving Pattern of Mobile Sink

According to moving pattern of the mobile sink, these algorithms are classified into two categories: mobility without purpose and mobility with purpose. In the first case, sink moves without purpose, which means sink moves randomly [25,26,27,28,38,39] or along a predetermined trajectory (e.g., Hilbert curve, roundness curve, square curve, and so on) [14,24,29,40,41,42]. In the other case, sink’s movement can be influenced by parameters of current network [3,16,17,18,19,20,22,32,43].

#### 2.4.1. Mobility without Purpose Pattern

We talk about two conditions in this case. The first is that sink moves randomly in network. In [25], proposed by Shin et al., sink moves randomly. To make source data follow sink’s steps, milestone node is introduced. No matter sink moves straight or changes its direction, milestone nodes can help sensory data to be routed to the sink. In algorithm of [26], source nodes establish virtual grid structure, and sink moves randomly to collect data. In Ring routing [27], proposed by Tunca et al., the sink just needs to tell its current location to the ring structure. Then data always can be routed to the sink which moves randomly. In this condition, there is no process of planning moving trajectory, hence the system maintenance is very simple. However, the sink may move to areas where much energy has been consumed or areas where source nodes are rare. In the second condition, the sink moves along a predetermined trajectory. In some algorithms, the sink moves around a boundary of network area. In BTDGS, the sink moves along the boundary of the circular network area. The sink may park in each sector for a period of time. When the sink moves in to a sector, it will park in the sector until the count number reaches the threshold. The time of parking in each sector is always different. In [29], proposed by Chen et al., the network area is the same as BTDGS. The sink also moves around the boundary area, and the moving path is also a circle. The sink moves along the circle path to collect data. In VGDRA, the sink also moves around the boundary of the network area. In some other algorithms, sink traverses in network. In [41], proposed by Mottaghi et al., sink travels through the middle of network, and the trajectory is fixed. Sensory data will be routed toward this trajectory. In [42], proposed by Konstantopoulos et al., the algorithm proposed is used in the application which involves a set of isolated urban areas (e.g., urban parks or building blocks) covered by sensor nodes. Mobile sinks are placed on public transport with fixed trajectories to collect data. So the trajectory sink moves are fixed. Similar to [41,42], algorithm in [40] also adopts a fixed trajectory. A tree structure is created among nodes. Nodes will upload data toward the trajectory by the tree structure. The sink will park in each grid for some time. These algorithms all adopt fixed trajectory to collect data. This way can make trajectory planning easy. However, the trajectory is so fixed, that sink’s movement cannot adapt to the change of network.

#### 2.4.2. Mobility with Purpose Pattern

The algorithms in which sink moves without purpose generally have high stability and reliability, and the system maintenance is simple. However, the status of network is always changing. Hence these algorithms cannot have a good performance. That means the sink needs to plan its trajectory dynamically.

In [20], proposed by Kinalis et al., the network area is partitioned in equal square cells firstly. When the sink moves in the network, the next position of the sink is determined by selecting (with some appropriate probability) one of the neighbors of the current vertex/cell in the graph. Sink keeps a counter to count how many times the sink has visited one grid. Thus, the frequency of visiting of each area can be estimated and moving trajectory is flexible. It can make energy consumption more balanced. As the sink stops in the area with more nodes for a long time, it can also make whole energy consumption lower. So mobility with purpose makes sink adjust its movement trajectory dynamically when parameters of network are changed. For example, when an event area is changed to another area, then the sink will move toward the new area.

### 2.5. Summary

The advantages and disadvantages of each data collection algorithm are summarized in Table 1. In this paper, we take the situation of event area changing and time delay into account. Our target application scenario is an event-driven network. Hence, in our GSDCS, we adopt active uploading and virtual grid structure.

## 3. Greedy Scanning Data Collection Strategy

### 3.1. Network Model

**Figure 2:**
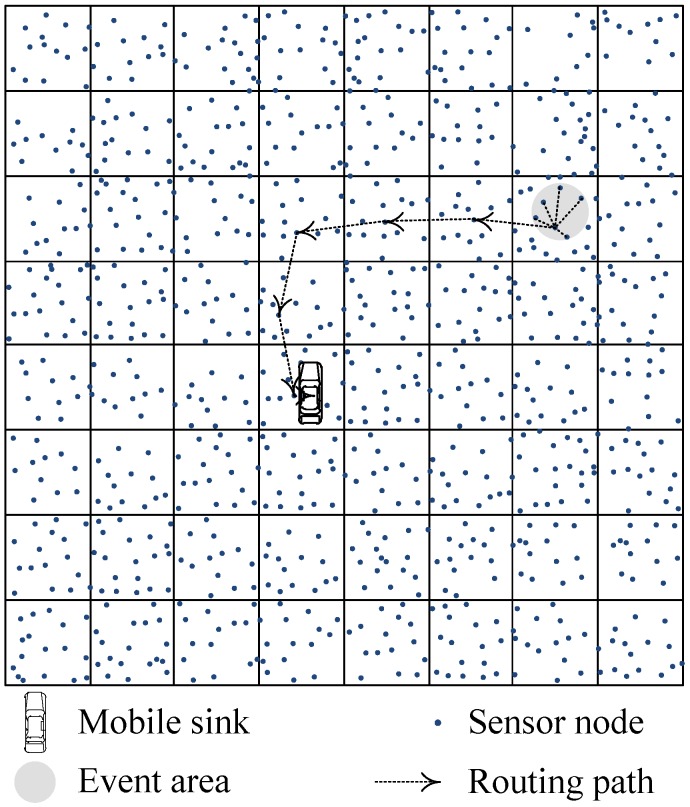
A wireless sensor network with virtual grid structure.

Data gathering strategies using mobile sink can prolong the network lifetime. However, for event area changing continuously situation, normal data gathering strategies with mobile sinks are not suitable. Especially, when the events involve animals migrating, enemy troops’ intrusion, and fire monitoring of forest, the events area appears dynamically, and regionally. Our GSDCS is designed for this situation. The typical application for this situation is animals monitoring. In this kind of application scenario, the wild animals have many group behaviors in many cases [44,45,46]. Hence, we set our application scenario as social animals monitoring in a field. Then the event area is local, so we use a circular to represent the event area. As the animals may migrate or interest of monitoring may change, the location of circular event area may change with time. As illustrated in Figure 2, sensor nodes are deployed randomly in a rectangle area. The network is divided into grids, consists of *N* nodes, and one mobile sink, which collects data from the whole network. The number of virtual grid cells are *M*. All nodes are well connected, and the network is full covered. Sensor nodes are static and location aware (i.e., equipped with GPS-capable antennae). Each node has the same initial energy, sensing radius $r_{s}$, and communication radius *R*. To guarantee the full cover of network, $N\geq Ma^{2}/\pi r_{s}^{2}$ is necessary. However, in the simulation or practical application, the *N* adopted is much larger than $Ma^{2}/\pi r_{s}^{2}$. Hence, we assume that each virtual grid cell always has sensor nodes. The mobile sink is not constrained by energy and moves without predefined trajectory. The trajectory of mobile sink is constructed dynamically according to the current status of network. The situations of obstacle or multi-path are not discussed in this paper. In our GSDCS, the notation and corresponding definition is shown in Table 2.

Our GSDCS is composed of three phases:

- Network initialization: To achieve the goal of updating sink location in a local area, the network area is divided into virtual grids. Nodes in the same grid form a group. The initialization process includes the grid structure establishment, head nodes election and neighbor table establishment. The details are discussed in Section 3.2.
- Data collection: In this phase, the way of data routing is proposed first. With the cooperation of virtual grid structure, this data routing way can deliver sensory data to the mobile sink easily. Whereafter, we talk about the moving pattern of the mobile sink with which energy consumption of network can be balanced. To minimize the energy cost in updating sink location information, the local updating with virtual grid structure is discussed at the end of this phase.
- Head nodes re-election: The long time working of head nodes may lead to network energy being unbalanced and lifetime being decreased. To improve the performance of the network, the re-election of head nodes is discussed in Section 3.4.

### 3.2. Network Initialization

As described in Section 3.1, after the network deployment, the first phase is initializing the network. It includes three sub-phases, the establishment of grid-based virtual structure, the election of head nodes and the establishment of a neighbor table.

#### 3.2.1. The Establishment of Virtual Grid Structure

As the deployment area is a rectangle, we adopt Cartesian coordinates for convenience. The origin of coordinates is located at deployment area center. The sink is static during the establishment. The process of establishing the virtual grid structure is as follows:

##### *Step 1. Calculate the Virtual Grid Cell Side Length* 

**Figure 3:**
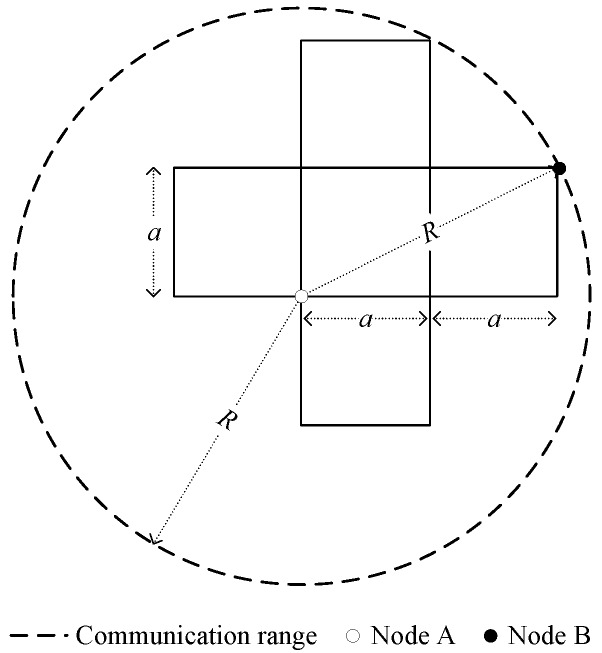
The relation of communication radius with side length of grid cell.

In order to guarantee that each node can communicate with all nodes in its neighbor grids (i.e., UDLR of a grid), The relation of grid cell side length *a* and node communication range *R* must satisfy $a\leq \sqrt{5}R/5$, as shown in Figure 3. In our simulation, we adopt the *a* as $\sqrt{5}R/5$ for energy efficient purpose.

##### *Step 2. Broadcast the Sink Initial Location and Grid Cell Side Length* 

**Figure 4:**
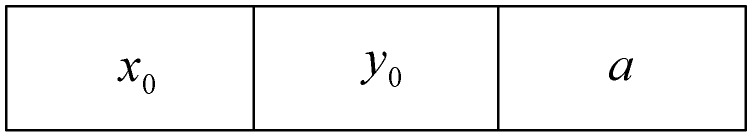
Format of HELLO packet.

After calculating the grid cell side length, the mobile sink broadcasts a HELLO packet to all nodes, which includes the side length of grid *a* and the coordinate of sink. The initial location of sink is $\left(x_{0},y_{0}\right)$. The HELLO packet is shown in Figure 4.

##### *Step 3. Calculate the RCN for Every Grid Cell* 

**Figure 5:**
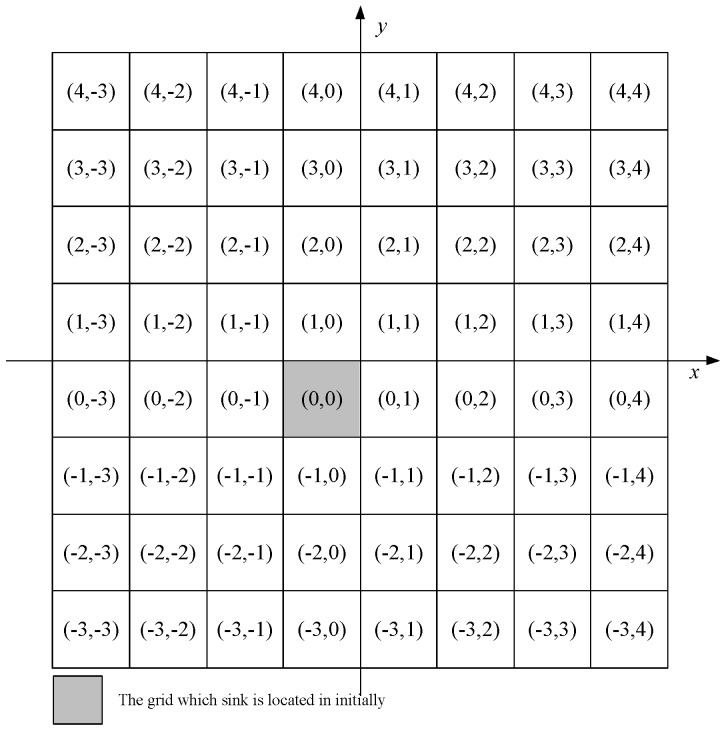
The row column number (RCN) of each virtual grid cell.

To identify each grid cell and their relative position in the deployment area, we introduce RCN (Row Column Number), which is composed of row number ($M_{r}$) and column number ($M_{c}$), as illustrated in Figure 5. For each node *i* can compute the RCN it belongs to by Equations (4) and (5) based on its location $\left(x_{i},y_{i}\right)$ (1≤i≤N), and the broadcast HELLO package received in Step 1. As all nodes are static, the RCN of each grid cell is determined and unchangeable after the network is deployed.

(3) $$RCN=(M_{r},M_{c})$$

(4) $$M_{r}=\left\lceil \frac{y_{i}-y_{0}-\frac{a}{2}}{a}\right\rceil$$

(5) $$M_{c}=\left\lceil \frac{x_{i}-x_{0}-\frac{a}{2}}{a}\right\rceil$$

##### *Step 4. Calculate the DN for Every Grid Cell* 

**Figure 6:**
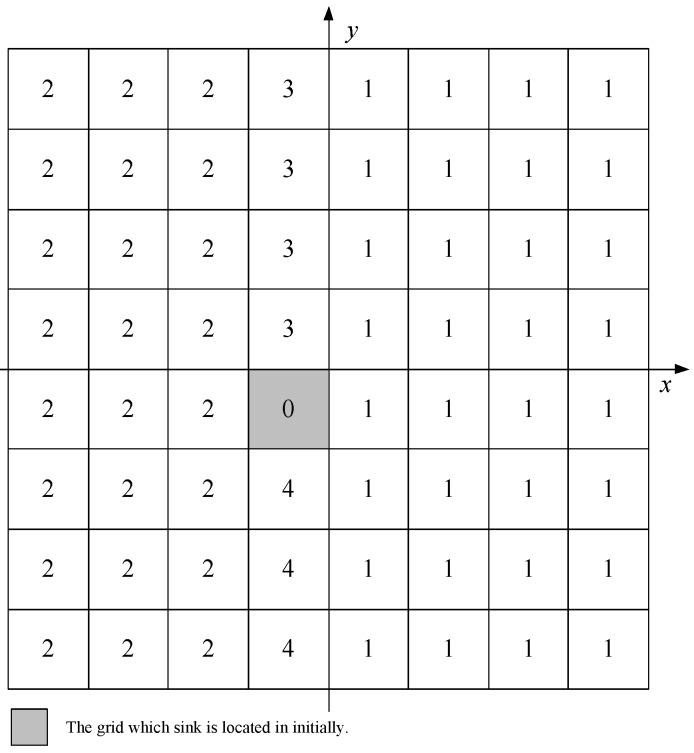
The direction number (DN) of each grid cell.

The mobile sink is moving from one grid to another during the data collection. To reduce the location broadcasting scope, each grid cell calculates the relative direction to the mobile sink. We introduce the concept DN (Direction Number) for each grid cell to describe the relative direction, as shown in Figure 6. The grid cell in which mobile sink is located is marked as GS (Grid of Sink). The value of DN and its meaning are as follows:

1. The current grid cell is the GS;
2. The current grid cell is located in the right side of column that GS belongs to;
3. The current grid cell is located in the left side of column that GS belongs to;
4. The current grid cell is right above GS;
5. The current grid cell is right under GS;

Each node computes its DN according to the HELLO packet. The process of calculation is shown in Equation (6).

(6) $$DN=\left\{\begin{array}{cc} 0 & ,\;\left|x_{i}-x_{0}\right|\leq \frac{a}{2}\&\left|y_{i}-y_{0}\right|\leq \frac{a}{2}\;\\ 1 & ,\;\left(x_{i}-x_{0}\right)>\frac{a}{2}\\ 2 & ,\;\left(x_{i}-x_{0}\right)<-\frac{a}{2}\\ 3 & ,\;\left|x_{i}-x_{0}\right|\leq \frac{a}{2}\&\left(y_{i}-y_{0}\right)>\frac{a}{2}\\ 4 & ,\;\left|x_{i}-x_{0}\right|\leq \frac{a}{2}\&\left(y_{i}-y_{0}\right)<-\frac{a}{2} \end{array}\right.$$

As the DN indicates the relative position between the current grid cell and the mobile sink, the DNs of some grid cells will be modified when the mobile sink moves into another grid cell.

#### 3.2.2. The Election of Head Nodes

It will cause significant energy by broadcasting the mobile sink new location to every node, routing the sensory data to the sink hop by hop. In our GSDCS, the concept “head nod” is introduced, it has three tasks: (1) collecting sensory data from the same grid cell nodes; (2) routing the collected data and the packets from neighbor head nodes to the mobile sink; (3) maintaining the DN value of its current grid cell when the latter is moving. After the mobile sink moves into a new grid, it informs some grid cell head nodes to update their DN value.

Two parameters are used to determine which nodes can be chosen as the head nodes: (1) residual energy of each node; (2) distance between each node and center point of corresponding grid cell. The node which has more energy and shorter distance is more suitable to be a head node in the same grid cell. Here we have Equation (7). Each node broadcasts its *K* value in the current grid cell. The node with a minimum *K* is elected as the current grid cell head node.

(7) $$K=\frac{D_{ToCenter}}{E_{residual}}$$

#### 3.2.3. The Establishment of Neighbor Table

After each grid cell has elected its head node, these head nodes need to establish their own neighbor table. Firstly, these head nodes broadcast their RCNs, and coordinates in their communication range. As described in Section 3.2.1, all the head nodes in neighbor grids can receive the information. According to the broadcast information, neighbor head nodes can be classified into four categories, as shown in Table 3. From the neighbor table, each head node can get the neighbor head node’s coordinate in corresponding direction. For example, if a head node is in the left neighbor grid, its coordinate will be written in the column of LG.

We assume that the RCN of a head node is $\left(M_{r},M_{c}\right)$ and the RCN of its a neighbor head node is $\left(M_{r}^{\prime },M_{c}^{\prime }\right)$. The criterion of classification for neighbor head nodes is shown in Table 4.

### 3.3. Data Collection

In the phase of data collection, there involves to elements, the nodes and the mobile sink. Hence, we discuss this phase from two aspects: the data routing and the sink trajectory planning.

#### 3.3.1. Data Routing

**Figure 7:**
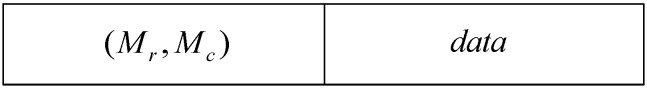
The data packet structure of sensory data.

The purpose of data routing is to deliver sensory data packets to the mobile sink from source nodes. When source nodes sense data, they encapsulate the sensory data and RCN of the source node together into a data packet, as shown in Figure 7. These data packets are routed to the sink hop by hop among head nodes.

When a node generates a data packet, it transmits the latter to its head node in the same grid cell. That means each head node collects data packets from source nodes which are firstly located in the same grid. Then, these head nodes transmit the data packets to a neighbor head node. After the neighbor head node gets the data packets, it relays the data packets to its neighbor head node until the DN of the current head node is 0. Finally, the head node transmits the data packet to the sink, and the transmission of source data is completed. By checking the DN, one head node can figure out the direction to transmission. Hence, based on the direction to transmission, the head node can locate the next hop neighbor head node from the neighbor table, as shown in Table 5.

**Figure 8:**
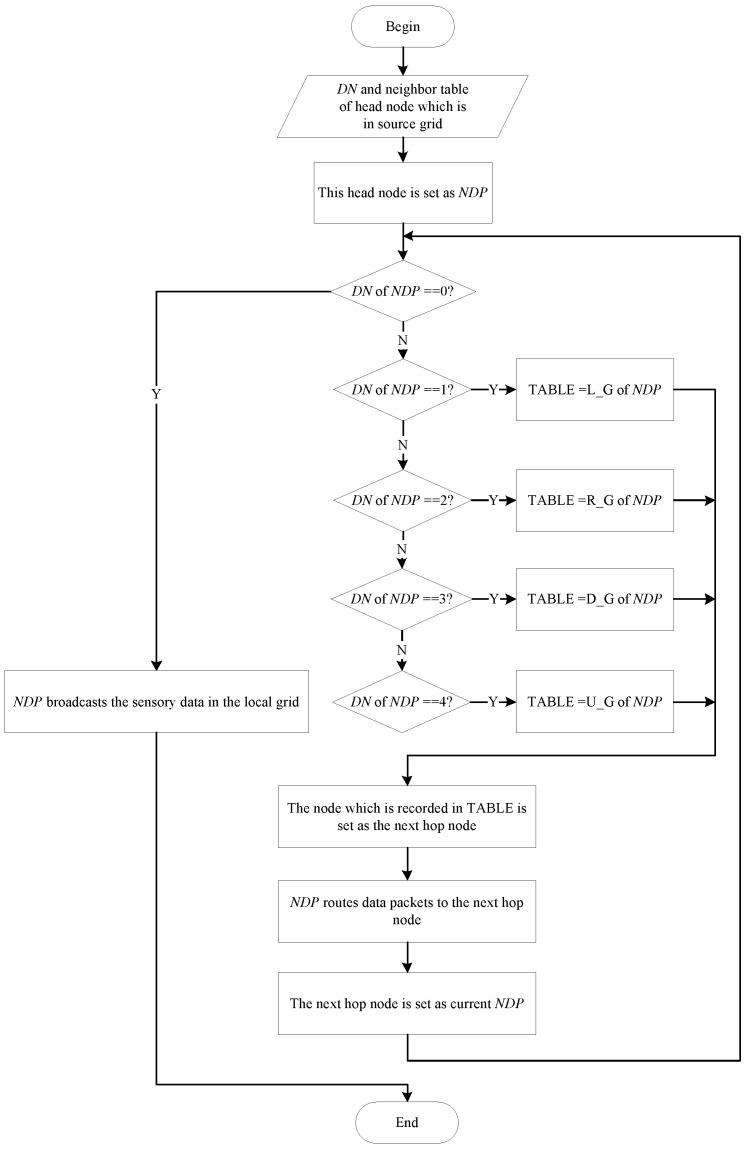
Flow chart of routing process.

For example, if the DN of a head node *A* is 1, then node *A* checks LG column of its neighbor table (this column records the coordinate of the left neighbor head node). We assume the node recorded in LG column is node *B*. Node *B* is designated as the next hop node, and the data packet is delivered to node *B*. When node *B* receives the data packet, it also checks its DN and executives the same procedure. Finally, the data packet will reach to node *X* which DN is 0. This condition indicates that node *X* and sink are in the same grid. Hence, node *X* uploads the data packet directly to the sink. The routing process can be described by the flow chart in Figure 8, and we assume the head node which holds the data packet is NDP for short.

#### 3.3.2. Sink Trajectory Planning

In our GSDCS, the mobile sink moves from one center point of a grid cell to another, and collects sensory data. As describe in previous subsection, each node routing the sensory data to the mobile sink. Hence, GSDCS needs to address two problems for mobile sink moving: (1) mobile sink moving pattern; (2) location information local updating.

**Figure 9:**
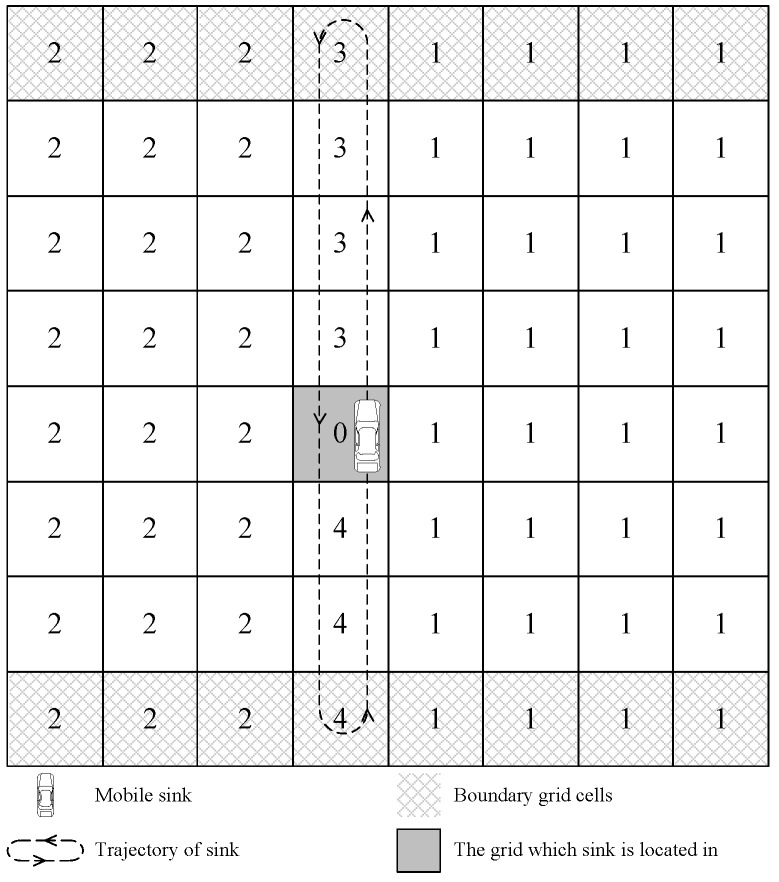
The trajectory of the sink in one collecting period.

In our strategy, the moving pattern of the mobile sink is designed like scanning curve in CRT (Cathode Ray Tube) screen. That is why our strategy is called scanning strategy. The deployment area is divided into grid cells, and the mobile sink traverses along one column as one round trip, which is called one collecting period. After each collecting period, the sink switches to another column to start its next round trip based on our greedy strategy. In one collecting period, the sink moves to grid cell whose DN is 3, firstly. When the sink moves to a new grid cell, it parks in the center of the new grid cell for a while. The mobile sink may collect data when it is moving. After the mobile sink has parked in a boundary grid cell for a while, the sink moves to the opposite direction, as shown in Figure 9. When the mobile sink arrives at another boundary grid, it changes its direction again. If the mobile sink arrives at the gird cell where it starts the collecting period, the collecting period ends.

Since the data packets contain the sensory data and the corresponding RCN, the mobile sink can count the total source nodes in each row, in the current column, in the current column left area, and in the current column right area, respectively. The mobile sink decides the column to move to in the next collecting period based on this counted information. The counted information is also used by the mobile sink to decide the parking time in each grid. If one row which a grid is in has more source nodes, the mobile sink will park in this grid for a longer time, dynamically.

We assume that the speed of the mobile sink is a constant *v*, and the longitudinal length of network is *L*. The time of moving in a period is T=2(L−a)/v. We also assume that the total time of static state for parking is equal to the total time for moving in a collecting period. Hence, the total time of a collecting period is 2T. In the first collecting period, the mobile sink spends the same time in each grid cell. The time sink spends in a grid consists of parking time and moving time. The total time sink spends in each grid is $2T/\left\lceil L/a\right\rceil$. When the sink has been in a non-boundary grid cell for $T/\left\lceil L/a\right\rceil$, it moves into the next one. After the sink has been in a boundary grid cell for $2T/\left\lceil L/a\right\rceil$, it also moves into the next one. The mobile sink maintains four kinds of counter, Counter(i), $Counter_{left}$, $Counter_{middle}$, $Counter_{right}$. The *i* represents $M_{r}$ of one row. As we have mentioned above, the data packet consists of RCN, and sensory data. $M_{r}$ and $M_{c}$ of RCN give information about which row and column the data packet comes from. When the mobile sink gets one data packet, it adds the corresponding counter, respectively. The Counter(i) can reflect how many data packets are collected from each row. The mobile sink can deduce the direction this data packet comes from: left side columns, right side columns or the current column.

When the first collecting period is completed, the mobile sink may switch the column based on our pervious description. It also needs to adjust the parking time in each grid cell according to the collecting period counter values.

**Figure 10:**
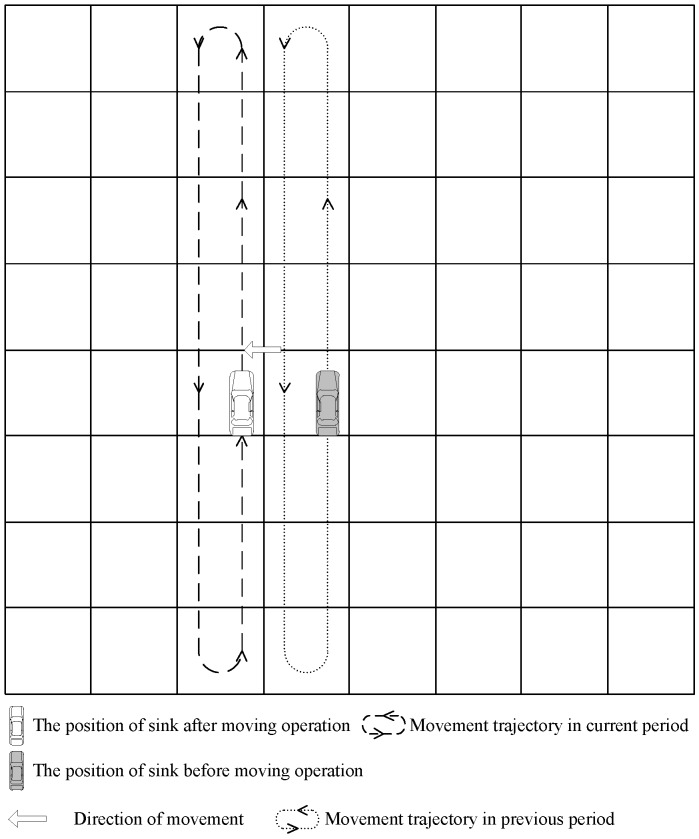
Sink moves to next column.

There are three choices for the sink: the left column, the current column and the right column. The mobile sink chooses the column $Cs=max(Counter_{left},Counter_{middle},Counter_{right})$ as its next collecting period column. When two or more counter values are equal, the mobile sink stays in the original column. The moving operation is shown in Figure 10.

As mentioned above, in the first collecting period, the parking time in each grid cell is equal. However, in real environments, obstacles and external disturbance may cause the distribution of sensor node and source events density uneven. Our GSDCS takes advantage of Counter(i) to adjust the parking time for each gird cell in the following collecting periods. If a grid cell has a larger Counter(i), the mobile sink will park in the grid for longer. Then the mobile sink calculates the ratio of the total data from one row to all rows, which can be formulated by $\frac{Counter(i)}{\sum Counter(i)}$. The adjustment the parking time of mobile sink in the grid cell of row *i* is shown as follows:

(1) If the grid of row *i* is a non-boundary grid, then the parking time in this grid is:
  (8) $$t_{park}\left(i\right)=\frac{T\times \frac{Counter(i)}{\sum Counter(i)}}{2}$$
  The total time sink spends in this grid for a single trip is:
  (9) $$t\left(i\right)=\frac{T\times \frac{Counter(i)}{\sum Counter(i)}}{2}+\frac{a}{v}$$
(2) If the grid of row *i* is a boundary grid, then the parking time in this grid is:
  (10) $$t_{park}\left(i\right)=T\times \frac{Counter(i)}{\sum Counter(i)}$$
  The total time sink spends in this grid is:
  (11) $$t\left(i\right)=T\times \frac{Counter(i)}{\sum Counter(i)}+\frac{a}{v}$$

**Figure 11:**
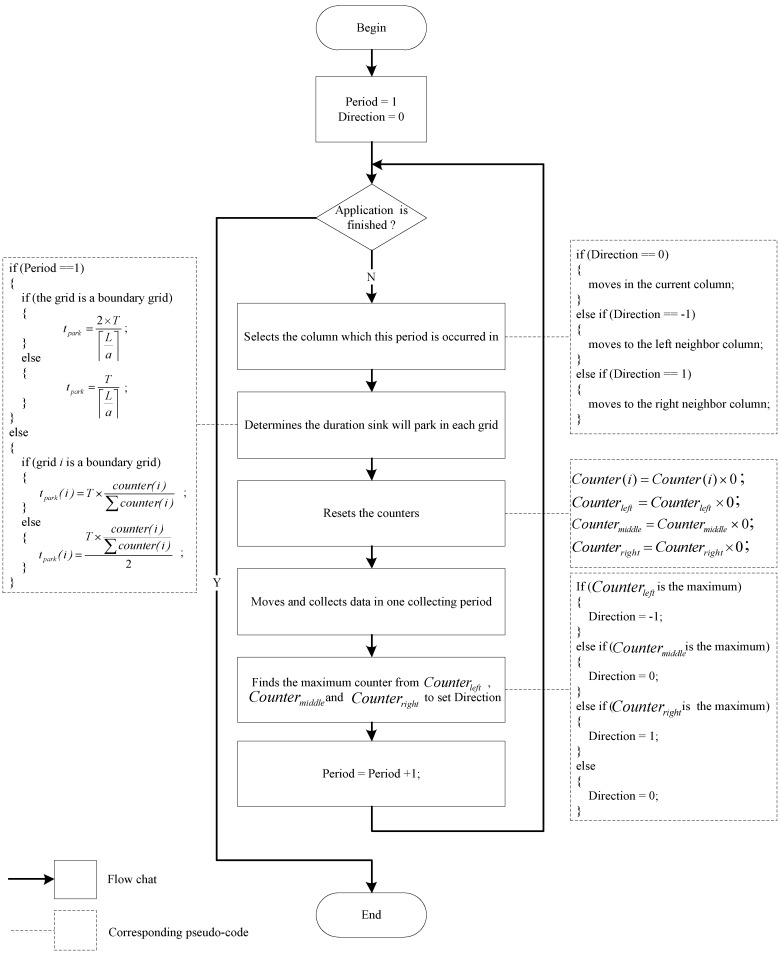
Flow chart of sink moving process.

Note, when a grid is a non-boundary grid, the mobile sink would travel through this grid twice in one collecting period. All the counters are set to zero at the beginning of a collecting period. When one collecting period completes, the mobile sink calculates the parameter t(i) of next period for each grid according to all the counters. The sink moving process can be described by the flow chart in Figure 11.

#### 3.3.3. Location Information Local Updating

**Figure 12:**
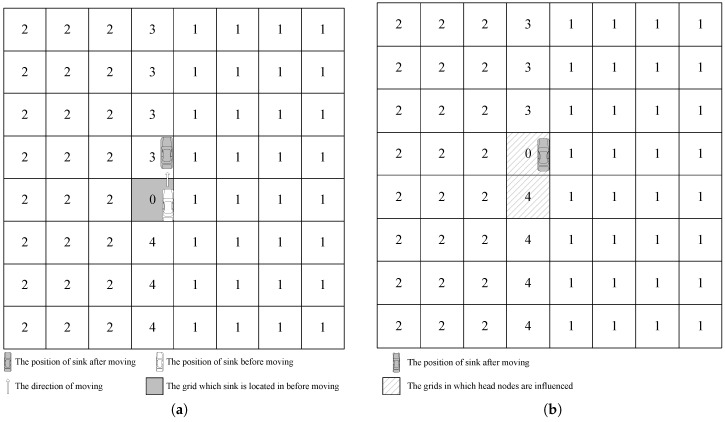
The way of updating when the mobile sink moves along the column in one collecting period. (**a**) The mobile sink moves to the upside grid cell; (**b**) The head nodes of the marked grid cells are needed to be updated.

To reduce the energy consumption for updating sink current location, our GSDCS only updates some head nodes’ DN when the mobile sink moves into a different grid. In our GSDCS, the DN is responsible for representing the relative direction of a grid to the mobile sink. Once the mobile sink moves into a new grid cell, it broadcasts a notification packet to the head nodes of corresponding grid cells for updating the DN value. There are two cases for updating the location information when the mobile sink moves into a neighbor grid cell: (1) the mobile sink moves along the column in one collecting period; (2) the mobile sink moves into another column when goes on to the next collecting period. If the mobile sink moves along the column in one collecting period, then two head nodes need to update their DNs. One head node is in the grid cell which the mobile sink used to be, the other is in the grid cell which the mobile sink parks in currently. The procedure can be described in Figure 12. The DNs of two head nodes in the marked grids are updated. That means only two nodes consume energy when the mobile sink moves to a new grid cell along the column direction.

**Figure 13:**
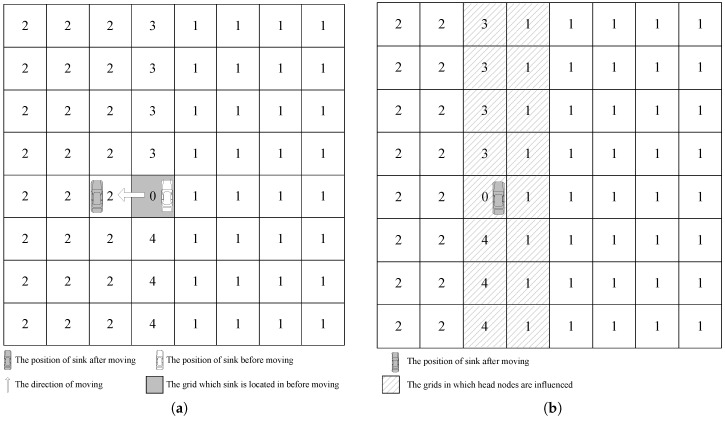
The way of updating when the mobile sink moves into another column when goes on to the next collecting period. (**a**) The mobile sink moves to the left neighbor column to start a new collecting period; (**b**) The head nodes of the marked grid cells are needed to be updated.

If the mobile sink moves into another column when goes on to the next collecting period, all the head nodes which are in the two columns need to update their DNs. One column is which sink used to be in. The other is the one sink parks in currently. This procedure can be described in Figure 13. The head nodes in the marked grid cells need to update their DNs. That means 16 head nodes consume energy when the mobile sink moves into another column in our example.

### 3.4. Head Nodes Re-Election

To achieve balanced energy consumption, the head node should be re-elected when its energy is below a certain threshold. Here we appoint $E^{\prime }$ as the current energy of the head node, while *E* is the original energy when it is elected as head node. If the ratio of $E^{\prime }$ to *E* is below a threshold Th, then the operation of re-election will be started. The current node informs other nodes in the same grid cell, and the latter compete for the role of head node with the new *K*. After a new head node is elected, it broadcasts in its communication range. The nodes in the same grid could know which one is the current head node. The relation of grid cell side length *a* and node communication range *R* must satisfy $a\leq \frac{\sqrt{5}R}{5}$ as described in Section 3.2.1, all the neighbor head nodes can receive this information. According to this broadcasting information, the neighbor head nodes are also informed about the change of head node in this grid. Then, these neighbor head nodes update their neighbor table, and the former head node transfers its DN and neighbor table to the current head node. In the re-election process, the node which is going to retire still has the duty of head node until the new elected node becomes a head node. That is to say, the old head node is still responsible for collecting and routing sensory data before the re-election process is completed.

## 4. Simulation and Performance Evaluation

### 4.1. Simulation Model

The simulation parameters and corresponding range of values are listed in Table 6. Then we talk about these parameters in detail. In our simulation, sensor nodes are deployed in a square area, and the side length of this area is 200 m. The number of nodes varies from 200 to 600. All nodes have the same communication range and initial energy. They are 75 m and 2 J respectively. The energy model in HEED [47] is adopted. To receive $n_{b}\;bits$ at the receiver, the radio expends $n_{b}\times E_{elec}\;J$. The energy consumption of transmitting $n_{b}\;bits$ at the sender has two cases. Here we define the distance between a sender and a receiver as *d*, and threshold distance of communication is $d_{0}$. In the first case, if $d<d_{0}$ is occurred, the sender consumes $n_{b}\times \left(E_{elec}+E_{fs}\times d^{2}\right)\;J$. If $d\geq d_{0}$, $n_{b}\times \left(E_{elec}+E_{mp}\times d^{4}\right)\;J$ is consumed. The value of $E_{fs}$ is 10 pJ/bit/m^2^, and $E_{mp}$ is 0.0013 pJ/bit /m^4^. The control message size is 25 bytes, and data packet size is 100 bytes. To simulate the uneven event source, we assume the event area is a circular area, and all the nodes in this area are appointed as source nodes. Sink moves at a constant velocity, and source nodes have a constant rate of producing sensory data. The lifetime of network is the time when the first node runs out of its energy. The performance metrics are as follows:

- Lifetime: The time when the first node runs out of its energy.
- Average Residual Energy: The average residual energy of all nodes when the network is ended.
- Length of Sink’s Movement: The length of sink’s trajectory when the network is end.
- Variance of Residual Energy: The variance of all nodes’ residual energy when the network is ended.
- Number of Data Packets Collected: The number of data packets collected by sink when the network is ended.

### 4.2. Performance Analysis under Different Parameters

#### 4.2.1. The Impact of Head Nodes’ Threshold Th

We first investigate the impact of head node re-election threshold on the performance of our algorithm by varying the value of Th. The parameters we set are shown as follow: (1) the number of all nodes is 500; (2) $r_{e}$ is 30 m; (3) the event area changes by period, and the period is 800 s; (4) one source node produces one data packet in one second; (5) the velocity of sink is 5 m/s; (6) Th can be 10%, 20%, 30%, 40%, 50%, 60%, 70%, 80% and 90%. As we mentioned in Section 3.4, $E^{\prime }$ as the current energy of the head node, while *E* is the original energy when it is elected as head node. If the ratio of $E^{\prime }$ to *E* is below a threshold Th, then head nodes will be re-elected.

**Figure 14:**
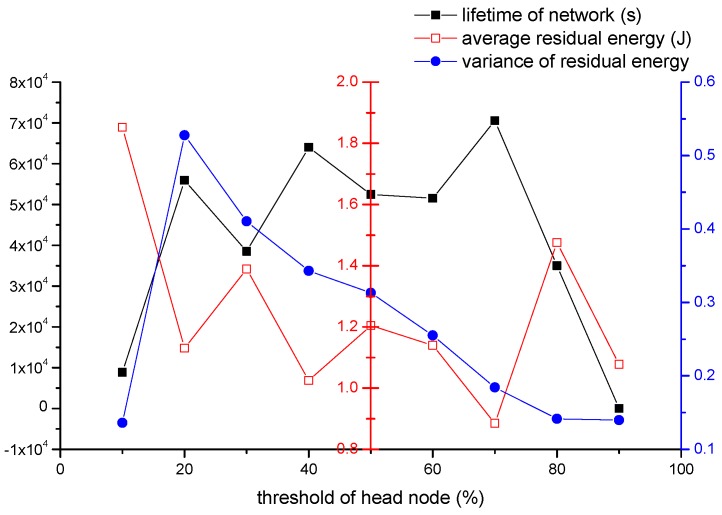
The impact of Th on the network performance.

In Figure 14, (1) black line represents the impact of Th on the lifetime of network. The network can obtain a relatively long lifetime when Th increased from 20% to 70%. A lifetime achieves the biggest value when Th is 70%. When Th is too low or too high, network dies very soon. According to the definition of Th, head nodes with a small Th consumes more energy when they retire. That means these retired head nodes have little energy while other nodes have much energy. These retired head nodes are easy to die. Network dies very soon with a small Th. In the opposite condition, if Th has a big value, re-election will be carried out frequently. As the re-election process consumes energy, hence energy is consumed fast. Then network also dies soon. When Th is in the middle area, the network has a relative balanced condition, which leads to a longer network lifetime.

(2) Red line represents the impact of Th on the average residual energy. When Th is 10%, the average residual energy is very high. As the analysis in (1), the end of network is just due to that some retired head nodes die soon. hence many nodes haven’t consumed much energy. So the residual energy is high. When Th increases, residual energy decreases with longer lifetime. When Th reaches 80%, re-election is carried out frequently, and residual energy increases with a decreased lifetime. When Th is 90%, much energy is spent on re-election process. The residual energy decreases although this condition has a short lifetime. The lower residual energy is, the better network runs. When Th is 70%, the residual energy is lowest.

(3) Blue line represents the impact of Th on variance of residual energy. The variance can reflect the balance of all nodes’ energy consumption. The network is more balanced when the variance is lower. At first, the variance is very low, that is because retired head nodes die so fast. When the first node dies, most nodes consume little energy. However, with the increase of Th, such as 20%, more nodes consume much energy while others consume little. The peak value is occurred in 20%. The variance is decreased from 20% to 90%. That is because the frequent re-election, and head nodes do not need to retire until much energy is consumed.

According to (1), (2) and (3), network can get the longest lifetime, the lowest average residual energy and a very low variance of residual energy when 70% is adopted.

#### 4.2.2. The Impact of Event Area’s Radius $r_{e}$

We study the impact of event area’s radius $r_{e}$ on the network performance. The event area’s radius increases from 20 m to 60 m. The parameters we set are shown as follows: (1) the number of all nodes is 500; (2) Th is 70%; (3) The event area changes by period, and the period is 800 s; (4) one source node produces one data packet in one second; (5) the velocity of sink is 5 m/s. The performance metrics here are network’s lifetime, residual energy’s variance and number of data packets collected.

**Figure 15:**
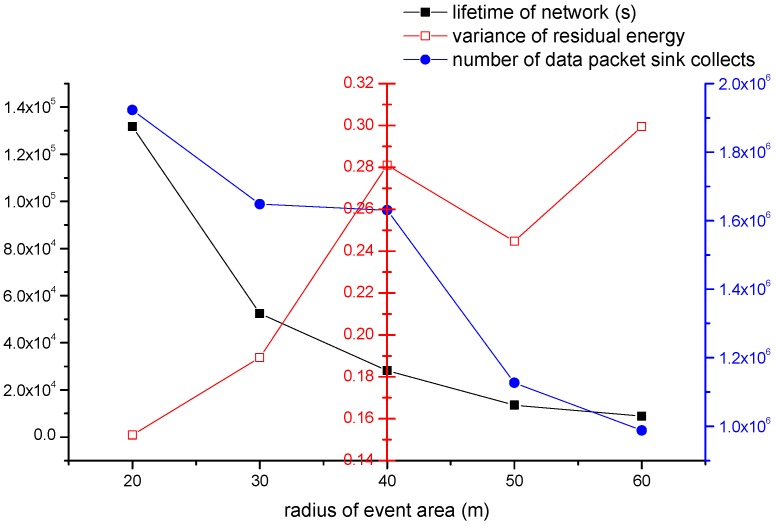
The impact of $r_{e}$ on the network performance.

In Figure 15, black line represents the impact of $r_{e}$ on lifetime of network. The network’s lifetime decreases by radius increasing. The network’s lifetime decreases fast firstly. Then speed of decrease becomes slow after that. As radius becomes larger, more nodes become source nodes. That means sink can collect more data packets in the same time. However, the lifetime of network is decreasing. The blue line represents the whole number of data collected in lifetime. We can find that the number of data packets collected is also decreased, and it decreases fast from 40 to 50. We can study the balance condition of network from red line. We can find that the red line is generally on the rise. Hence the network is balanced well when the radius is 20.

According to the three performance metrics, we can find that our algorithm can have a better performance with a small event area.

#### 4.2.3. The Impact of Period of Changing Event Area

In this section, we study the impact of period of changing event area. As we defined in Section 3.3.2, the time sink spend to complete a column running is 2T, so we appoint 2T as a unit to study this topic. We call 2T as column period. The parameters we set is shown as follow: (1) the number of all nodes is 500; (2) $r_{e}$ is 30 m; (3) Th is 70%; (4) one source node produces one data packet in one second; (5) the velocity of sink is 5 m/s. The period of changing varies from 2 to 16 column period.

**Figure 16:**
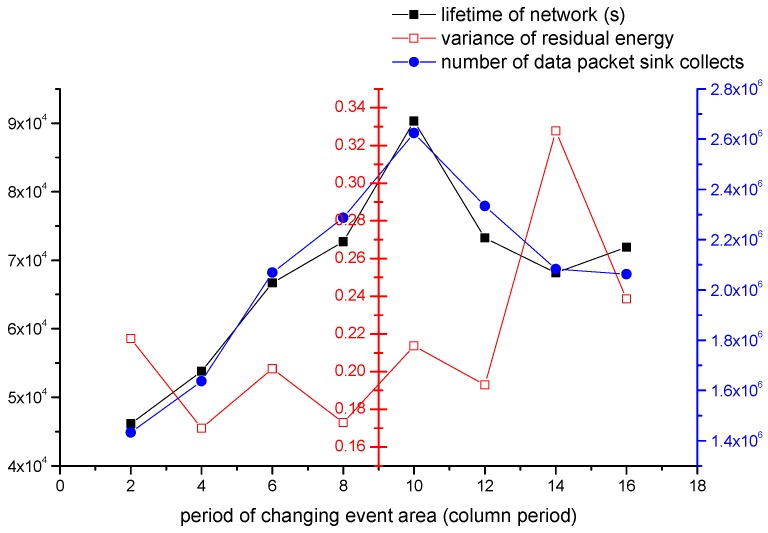
The impact of period of changing event area on the network performance.

In Figure 16, the black line represents the lifetime of the network. The network’s lifetime increases firstly, and it gets the peak value at 10. Then the network’s lifetime decreases. When the period of changing is 2 column period, the lifetime is the shortest. The blue line represents the number of data collected. The blue line is almost coincident with the black line. The red line represents variance of residual energy. Before 14 column period, the variance is relatively stable and small. When changing period is greater than or equal to 14 column period, variance increases fast. As we introduced in Section 3.3.2, sink has a trend to move to the area with more source data. The sink also has the trend to stay for longer in this area. Hence sink moves to event area in the experiment. According to the relationship between grid length and communication radius, our virtual structure has 6×6 grids. So when changing period adopts 2 column period, sink always can not reach the event area before event area changes. Energy couldn’t be saved and network dies soon. By the increasing of changing period, sink has sufficient time to reach event area and stays for more time in the event area. Then much energy can be saved and network works longer. We can find that network’s lifetime is longest when 10 column period is adopted. When changing period is greater than 10 period column, lifetime of the network decreases quickly. In this situation, event area stays in someplace for long time, and sink will always travel in the columns with source nodes. Although much energy is saved in overall, the nodes in the same columns with source nodes consume much energy. The nodes in other columns consume almost no energy. The network’s lifetime becomes shorter, and the variance of residual energy becomes higher. According to this experiment, the network can get a good performance when 10 column period is adopted.

#### 4.2.4. The Impact of Velocity of Source Data

In this section, we study the impact of velocity of source data on the performance of our algorithm. The parameters we set are shown as follows: (1) the number of all nodes is 500; (2) $r_{e}$ is 30 m; (3) Th is 70%; (4) the event area changes by period, and the period is 800 s; (5) the velocity of sink is 5 m/s. The number of data packets source nodes produce varies from 0.2 to 1.8 in one second.

**Figure 17:**
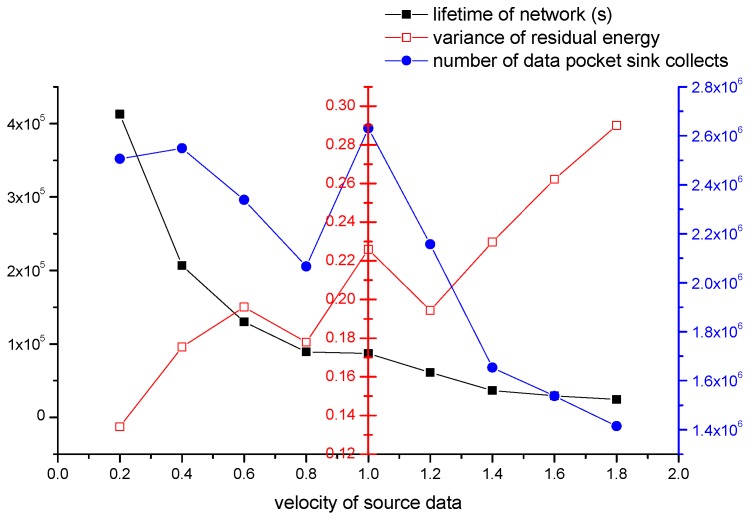
The impact of velocity of source data on the network performance.

In Figure 17, the black line represents network’s lifetime. The network’s lifetime decreases with the increasing of velocity of source data. When velocity is less than or equal to 0.8, network’s lifetime decreases fast. When velocity is greater than 0.8, network’s lifetime decreases very slowly. The blue line represents the number of data packets collected. The number is decreased in the mass. However, the number gets the peak value when velocity is 1.0. That means sink gets the most data although the lifetime of this situation is short. The red line represents the variance of residual energy. The red line is increased in the mass, so the network could be more balanced if the velocity is smaller.

According to the experiment results and analysis above, network can achieve a better performance if velocity is smaller in the mass. There is a special case in it. When velocity adopts 1.0, sink can collect the most data although the lifetime is short.

#### 4.2.5. The Impact of Velocity of Sink

**Figure 18:**
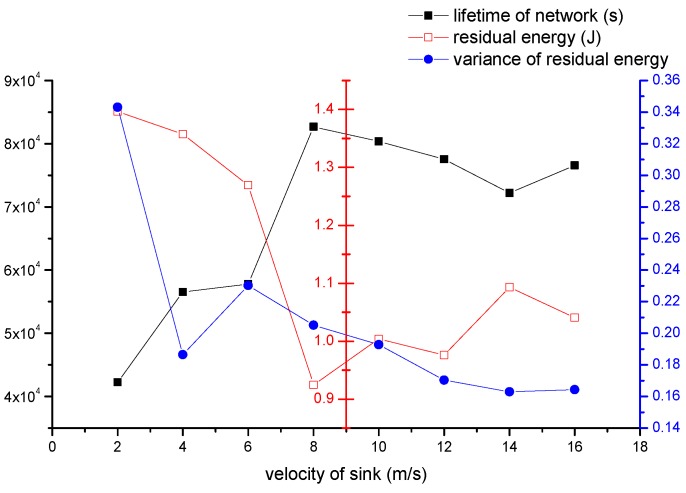
The impact of velocity of sink on the network performance.

In this section, we study the impact of velocity of sink on the performance of network. The parameters we set are shown as follows: (1) the number of all nodes is 500; (2) $r_{e}$ is 30 m; (3) Th is 70%; (4) the event area changes by period, and the period is 800 s; (5) one source node produces one data packet in one second. The velocity of sink varies from 2 m/s to 16 m/s. The result of experiment is shown in Figure 18.

The black line represents network’s lifetime. Network’s lifetime increases fast at first, and it gets a peak value at 8 m/s. Then network’s lifetime decreases slowly. In our algorithm, if sink moves slowly, then nodes around sink may consume much energy. Hot spot problem will occur. If sink moves fast, the network needs to update frequently. The operation of updating also consumes energy, and the energy consumed of updating is much less than energy consumed of transmitting data, thus there will be a point where the most balanced situation is achieved, and 8 m/s is the point. Network’s lifetime increases fast from 2 m/s to 8 m/s. That is because when sink moves faster, the energy consumption will not be always concentrated in a region. Hence network is becoming more and more balanced, and lifetimes becomes longer. When more than 8 m/s, there is a slight decline. That is the impact of too frequent updating operation. The red line represents average residual energy. Corresponding with the black line, average residual energy is low when network’s lifetime is high. The blue line represents the variance of residual energy, and it is decreased in the mass. That means the network is more balanced when sink moves faster.

According to the data obtained from this experiment, the network gets the best performance at 8 m/s, and the performance of network is relatively good when velocity of sink is greater than 8 m/s.

### 4.3. Comparison with VGDRA

**Figure 19:**
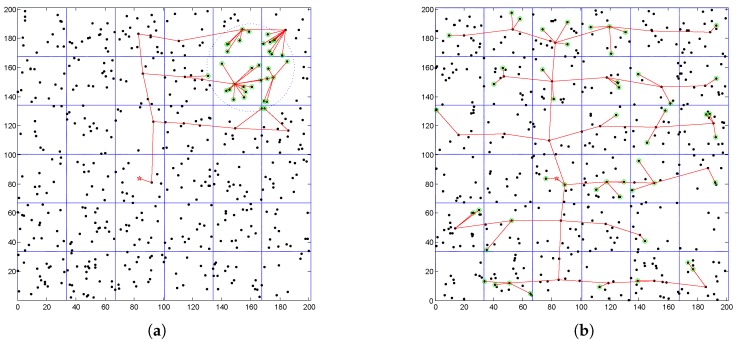
Two application scenarios (**a**) Source nodes are distributed in a local area; (**b**) Source nodes are evenly distributed.

In this section, we compare the performance of proposed strategy with VGDRA [24]. VGDRA also adopts virtual grid structure and mobile sink, but sink’s trajectory is fixed. We compare the two algorithms from three parts. Part 1: The source nodes are distributed unevenly in a small area of network, as we can see in Figure 19a; Part 2: The source nodes are evenly distributed in the network, as we can see in Figure 19b; Part 3: Only the energy consumption of updating routes is considered. In the Figure 19, the red lines indicate the routing path. The dots with green border are source nodes. The red star is the mobile sink.

#### 4.3.1. Source Nodes Are Distributed Unevenly in the Network

**Figure 20:**
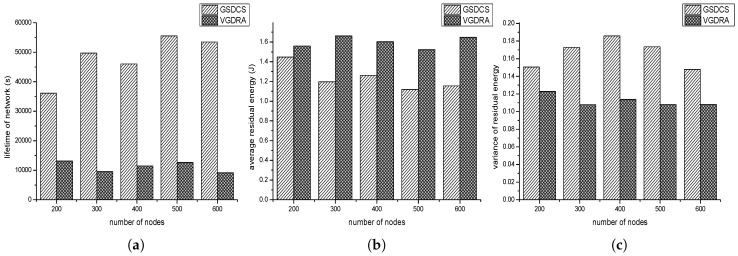
The comparison with VGDRA when source nodes are distributed unevenly. (**a**) Lifetime of network vs. the number of nodes; (**b**) Average residual energy vs. the number of nodes; (**c**) Variance of residual energy vs. the number of nodes.

In this part, the application scenario in which source nodes are distributed in a small area of network is set. The parameters we set is shown as follow: (1) the number of nodes varies from 200 to 600; (2) $r_{e}$ is 30 m; (3) Th is 70%; (4) the event area changes by period, and the period is 800 s; (5) one source node produces one data packet in one second; (6) the velocity of sink is 5 m/s. Figure 20a shows the impact of nodes’ number on the lifetime of network. As expected, the lifetime of our strategy is far greater than VGDRA’s. That is because the sink in our strategy can move toward the event area. However, in VGDRA, sink always moves along the fixed trajectory no matter where the event area is. The network of our strategy gets the maximum value when nodes number is 500. With the increase of nodes’ number, the lifetime of VGDRA is almost unchanged. Hence in this application scenario, the number of nodes has little impact on VGDRA’s lifetime. Figure 20b shows the impact of nodes’ number on average residual energy. Our strategy’s average residual energy is lower than VGDRA’s. Our strategy can make the network have a higher use ratio in this application scenario. Figure 20c shows the impact of nodes’ number on residual energy variance. Our strategy’s variance is little higher than VGDRA’s. According to the data of VGDRA’s network lifetime, VGDRA’s lower variance is due to that most nodes have not started working yet when the first node runs out of its energy. So our strategy has a better performance in this application scenario.

#### 4.3.2. Source Nodes Are Distributed Evenly in the Network

**Figure 21:**
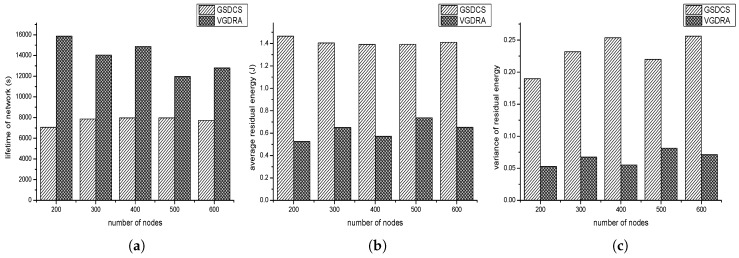
The comparison with VGDRA when source nodes are evenly distributed. (**a**) Lifetime of network vs. the number of nodes; (**b**) Average residual energy vs. the number of nodes; (**c**) Variance of residual energy vs. the number of nodes.

In this application scenario, source nodes are evenly distributed. Each place has the same probability of source node. The parameters we set is shown as follow: (1) the number of nodes varies from 200 to 600; (2) the source nodes to all the nodes ratio is 20%; (3) Th is 70%; (4) the event area changes by period, and the period is 800 s; (5) one source node produces one data packet in one second; (6) the velocity of sink is 5 m/s. Figure 21a shows the impact of nodes’ number on the lifetime of network. Figure 21b shows the impact of node’ number on the average residual energy. In our strategy, much energy hasn’t be used when network stops to work. The residual energy is wasted. From this perspective, VGDRA has a better performance. Figure 21c shows the impact of nodes’ number on the residual energy variance. We can find that our GSDCS’s variance is higher than VGDRA’s. When our strategy is used in this application scenario, the number of data packets sink collected from left side and right side may be almost the same. Then sink may always moves in the middle columns of network. Nodes in these middle columns may have “hot spot” problem. However in VGDRA, sink always moves along the boundary of network, so VGDRA can make network balanced. So VGDRA can work better in this application scenario.

#### 4.3.3. Only the Energy Consumption of Updating Routes Is Considered

**Figure 22:**
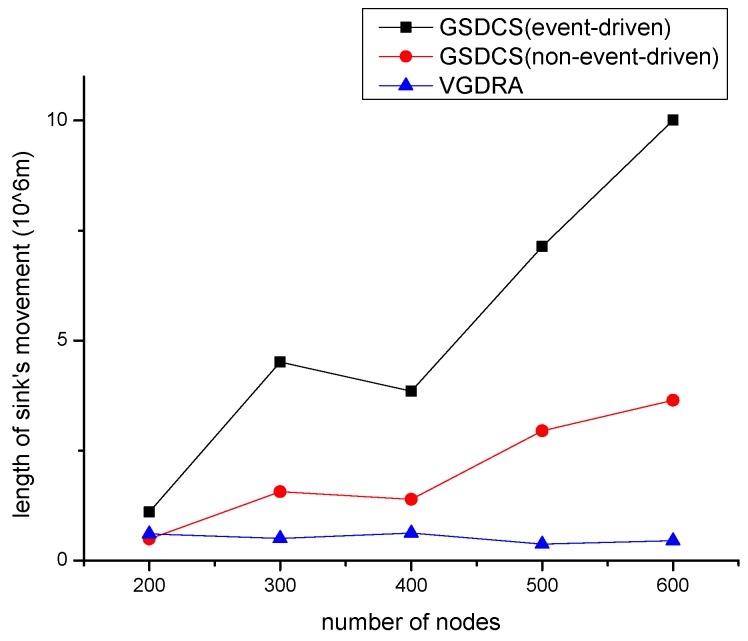
The comparison with VGDRA when only the updating energy is considered.

In this part, we study the cost of updating routes. We only deduct the energy consumption of updating routes. Here we adopt the initial energy in [24], and the initial energy is 1 mJ. As the distribution of source nodes has impact on results, so we talk separately. We use the length of sink moving as performance metric. That means we count how long sink has moved before the first node dies. Figure 22, shows the result of experiment. The blue line represents the length sink moves in VGDRA. The blue line is the lowest, and the length is almost unchanged with the increasing of nodes. The red line is the case of our GSDCS in an evenly distribution application scenario. The red line is higher than blue line, and the red line is increasing. The black line represents the lifetime of our GSDCS in the application scenario where source nodes are distributed in a small area of network. The black line is the highest, and it is increasing in mass. Hence, the cost of updating routes in our strategy is far below VGDRA’s.

## 5. Conclusions

In this paper, we have discussed the strategy of data collection with a mobile sink which was named GSDCS. We first proposed a virtual grid structure to assist data collection. Each grid cell was given RCN (row column number) and DN (direction number). Based on the structure, a way of data routing was proposed later. Combined with RCN and DN, data packets could be routed to sink easily. Then we proposed the trajectory planning of mobile sink. In the sink trajectory planning, sink has a tendency to move to the event area where most source nodes are located. By doing so, the total length of routing paths is shortened. Finally, to make the network more balanced, we proposed a way to re-elect the head node in each virtual grid cell. Compared with VGDRA in the simulation, GSDCS has a better performance in specific application scenarios.

## Figures and Tables

**Table 1:** Related work summary.

Categories		References	Advantages	Disadvantages
Uploading pattern	Passive uploading	[3,4,16,17,18,19,20,21,22,23]	Lower energy consumption	(1) Long latency of data collection (2) High pressure on memory of nodes
	Active uploading	[14,24,25,26,27,28,29]	Real time	(1) Hard to update sink’s current location quickly with lower energy consumption (2) Fast energy consumption around sink
Organization pattern	Non-virtual structure based	[16,25,30]	Network model is easy to established	The way to find current location of sink is inefficient
	Virtual structure based	[14,20,24,26,27,28,29,31,32,33,34,35]	(1) Nodes are well organized (2) Source nodes are easy to get sink’s location	Maintenance of some virtual structure is complex
Moving pattern	Mobility without purpose	[14,24,25,26,27,28,29,38,39,40,41,42]	Simple system maintenance	Cannot adapt to the dynamic changes of network
	Mobility with purpose	[3,16,17,18,19,20,22,32,43]	Good flexibility	Maintenance of system is not simple

**Table 2:** Notations and definitions.

Notation	Definition
$r_{s}$	Sensing radius of nodes
*a*	Side length of a grid cell
*v*	Speed of the mobile sink
*N*	Number of nodes deployed in network
*R*	Communication radius of nodes
*L*	Longitudinal length of network
$\left(x_{0},y_{0}\right)$	Initial coordinate of the mobile sink
$\left(x_{i},y_{i}\right)$ (1≤i≤N)	Coordinate of node deployed in network

**Table 3:** Neighbor table.

UG	coordinate of the upside neighbor head node
DG	coordinate of the downside neighbor head node
LG	coordinate of the left side neighbor head node
RG	coordinate of the right side neighbor head node

**Table 4:** The criterion of neighbor table establishment.

Criterion/Condition	Category
$M_{r}^{\prime }=M_{r}+1\&M_{c}^{\prime }=M_{c}$	UG
$M_{r}^{\prime }=M_{r}-1\&M_{c}^{\prime }=M_{c}$	DG
$M_{r}^{\prime }=M_{r}\&M_{c}^{\prime }=M_{c}-1$	LG
$M_{r}^{\prime }=M_{r}\&M_{c}^{\prime }=M_{c}+1$	RG

**Table 5:** The way to choose a neighbor head node.

DN Value	Direction to Transmission	Column Selected in Neighbor Table
1	left	LG
2	right	RG
3	down	DG
4	up	UG
0	(broadcast in local grid)	(broadcast in local grid)

**Table 6:** Simulation parameters.

Parameters	Definition	Default Settings
*L*	Side length of deployment area	200 m
*N*	Number of sensor nodes	200∼600
$r_{c}$	Node communication radius	75 m
$r_{e}$	Radius of event area	20∼60 m
*E*	Initial energy of each node	2 J
$d_{0}$	Threshold of communication	75 m
$E_{elec}$	Digital electronics	50 nJ/bit
$E_{fs}$	Communication $\left(d<d_{0}\right)$	10 pJ/bit/m^2^
$E_{mp}$	Communication $\left(d\geq d_{0}\right)$	0.0013 pJ/bi/m^4^
